# No Evidence of Complementary Water Use along a Plant Species Richness Gradient in Temperate Experimental Grasslands

**DOI:** 10.1371/journal.pone.0116367

**Published:** 2015-01-14

**Authors:** Dörte Bachmann, Annette Gockele, Janneke M. Ravenek, Christiane Roscher, Tanja Strecker, Alexandra Weigelt, Nina Buchmann

**Affiliations:** 1 Institute of Agricultural Sciences, ETH Zurich, Zurich, Switzerland; 2 Faculty of Biology, Department of Geobotany, University of Freiburg, Freiburg, Germany; 3 Department of Experimental Plant Ecology; Institute for Water and Wetland Research; Radboud University Nijmegen, Nijmegen, The Netherlands; 4 UFZ, Helmholtz Centre for Environmental Research, Department of Community Ecology, Halle, Germany; 5 J. F. Blumenbach Institute of Zoology and Anthropology, Georg August University Göttingen, Göttingen, Germany; 6 Department of Special Botany and Functional Biodiversity, Institute of Biology, University of Leipzig, Leipzig, Germany; 7 German Centre for Integrative Biodiversity Research (iDiv) Halle-Jena-Leipzig, Leipzig, Germany; WSL Institute for Snow and Avalanche Research SLF, SWITZERLAND

## Abstract

Niche complementarity in resource use has been proposed as a key mechanism to explain the positive effects of increasing plant species richness on ecosystem processes, in particular on primary productivity. Since hardly any information is available for niche complementarity in water use, we tested the effects of plant diversity on spatial and temporal complementarity in water uptake in experimental grasslands by using stable water isotopes. We hypothesized that water uptake from deeper soil depths increases in more diverse compared to low diverse plant species mixtures. We labeled soil water in 8 cm (with ^18^O) and 28 cm depth (with ²H) three times during the 2011 growing season in 40 temperate grassland communities of varying species richness (2, 4, 8 and 16 species) and functional group number and composition (legumes, grasses, tall herbs, small herbs). Stable isotope analyses of xylem and soil water allowed identifying the preferential depth of water uptake. Higher enrichment in ^18^O of xylem water than in ²H suggested that the main water uptake was in the upper soil layer. Furthermore, our results revealed no differences in root water uptake among communities with different species richness, different number of functional groups or with time. Thus, our results do not support the hypothesis of increased complementarity in water use in more diverse than in less diverse communities of temperate grassland species.

## Introduction

Many results from experimental biodiversity research support the hypothesis that increased plant species richness has positive effects on several aspects of ecosystem functioning [1–4], such as plant biomass production aboveground [5–8], whereas the underlying mechanisms for these positive effects are not yet fully understood [9]. One frequently proposed explanation is niche complementarity [10, 11], assuming that partitioning of resources such as light, nutrients or water reduces competitive interactions among the species of a mixture. Consequently, resource exploitation at the community level is more complete, resulting in greater productivity compared to a monoculture or low diverse mixture. Partitioning of belowground resources might be achieved spatially via different root distribution patterns or temporally because of differences in phenology among species [12, 13]. However, experimental evidence, particularly for the resource water, is still sparse.

Furthermore, the hypothesis of complementary resource use was mainly tested indirectly, for instance by comparing aboveground biomass production in mixtures with values expected from monocultures [14–16] or by interpreting a more complete filling of available biotope space, i.e., soil depth and volume, indicated by increased vertical root biomass distribution with increasing species richness as greater complementarity [17–19]. In addition, complementary water use was suggested based on increased evapotranspiration rates in plant mixtures with increasing species richness [20] or based on lower δ^13^C values in mixtures compared to monocultures [21]. Although water is an important resource for plant performance, there is, to our knowledge, a lack of direct measurements to assess water partitioning in mixtures and to test complementarity in water use with increasing species richness under field conditions.

Stable water isotopes have often been applied to directly estimate the water source used by plants (e.g., water of different soil depths or even fog) and were used in many studies aiming to explain coexistence of plants in different natural ecosystems [22–27]. Potential water sources of co-occurring species were identified by comparing the natural abundance of oxygen or hydrogen isotopes in xylem water and soil water of different depths. As no isotopic fractionation occurs during water uptake, the isotopic signal of the xylem water reflects the signal of a plant’s water source [28, 29]. In herbaceous plants, it has been shown that the isotopic signal of xylem water in the root crown was the best indicator of the water source [30]. However, natural abundance analyses rely on a pronounced isotopic profile of soil water, which is often not given under field conditions [31]. More unequivocal results can be obtained by enriching the soil water at different depths with different stable water isotopes [32, 33].

Thus, we carried out a labeling experiment in the Jena Experiment [34] and applied water enriched in stable water isotopes (oxygen and hydrogen) at two different depths three times during the growing season 2011. The Jena Experiment is a large grassland biodiversity experiment with communities of varying species richness and functional group number, based on a pool of 60 temperate grassland species which greatly differ in their functional characteristics (grasses, legumes, tall herbs, small herbs). Based on the niche complementarity theory, we expected (i) uptake of water from distinctly different soil layers with increasing species richness or functional group number, and (ii) functional characteristics, i.e., functional group identity, to explain spatial and seasonal variations in water uptake patterns.

## Material and Methods

### Study site

The Jena Experiment is a large temperate grassland biodiversity experiment located in the floodplain of the Saale river near the city of Jena (Germany, 50°55’N, 11°35’E, 130 m a.s.l.), which was established in 2002 on a former arable field. Since the Jena Experiment field site is not subjected to nature conservation and our study did not involve endangered or protected species, no specific permission was required. The soil is a Eutric Fluvisol developed from up to 2 m thick fluvial sediments. Mean annual precipitation is 587 mm, mean annual temperature is 9.3°C [35]. The Jena Experiment consists of 82 plots with different plant species number (1, 2, 4, 8, 16 and 60 species) and functional group richness (1, 2, 3 and 4 functional groups), from a species pool of 60 species assigned to four plant functional groups (grasses, legumes, small herbs and tall herbs). The experimental plots are arranged in four blocks to account for a gradient in soil texture, ranging from sandy loam to silty clay with increasing distance from the river. All plots are weeded three times per year (April, June and September) and mown two times per year (June, September) to mimic the management of extensive hay meadows.

### Tracer application and field sampling

The tracer experiment was conducted at the start of the growing season (April) and during the regrowth after the first and the second mowing (June and September) 2011. The experiment was carried out on a subset of 40 plots, covering a species richness gradient with well-established 2, 4, 8 and 16 plant species mixtures (ten replicates per species richness level, list of mixtures in S1 Table). These plots were selected to be equally distributed among the experimental blocks and to cover a balanced distribution of all functional group richness levels in each species richness level. At each plot, three subplots were established (44 cm × 56 cm), each for one of the three labeling campaigns of the tracer experiment.

About five days before starting the tracer application, plant and soil samples were collected 10 cm next to the study plots to identify the natural abundance of ^18^O and ^2^H (later referred to as background samples). Using a soil auger of 1 cm diameter (Eijkelkamp, The Netherlands), soil background samples were taken at one plot per species richness level in each of the four blocks in 0–10, 10–20, 20–30 and 30–40 cm soil depth, resulting in a total of 64 background samples per campaign. Root crowns, the connection between above- and belowground tissues, of single plants were collected and immediately placed into 12 ml glass vials (Labco Limited, UK), sealed with a cap and parafilm, and frozen until cryogenic water extraction was carried out. In total, 49 root crown background samples, homogenously distributed along the species richness gradient and representing species of each functional group in each species richness level, were collected prior each campaign.

For the tracer experiment, labeled water (^1^H_2_^18^O, Sigma-Aldrich, Germany, and ^2^H_2_O, Euriso-top, France) was injected at the same subplot (44 cm × 56 cm), but in different soil depths (^1^H_2_^18^O at 8 cm and ^2^H_2_O at 28 cm depth). To achieve a homogenous tracer distribution within the subplots, injection points were arranged on a grid of seven horizontal lines, which had a distance of 8.7 cm. The injection points for the two depths were alternating along the lines with a distance of 10 cm. This resulted in 32 injection points for the upper and 31 injection points for the lower soil depth (Application scheme in S1 Fig.). Holes of 8 mm diameter were drilled down to the two target depths of 8 and 28 cm with a handheld automated drill during five days prior to labeling, and stabilized with wooden sticks.

The tracer solutions were created to achieve an enrichment of 400 ‰ for ^18^O (upper soil depth) and 800 ‰ for ^2^H (lower soil depth), based on the average soil water content of all plots measured a few days prior to labeling. Thus, the following tracer solutions were created and added to the soil water: 8’700 ‰ δ^18^O and 26’500 ‰ δ^2^H in April (18 to 19 April 2011), 12’100 ‰ δ^18^O and 33’000 ‰ δ^2^H in June (27 to 28 June 2011) and 15’500 ‰ δ^18^O and 39’000 ‰ δ^2^H in September (27 to 28 September 2011). The tracer solutions were applied at 20 subplots per day between 8 am and 4 pm. The respective tracer solution was applied at 3 cm (^18^O-enriched water) or 23 cm depth (^2^H-enriched water) within 30 min per depth, using a 3 mm diameter four-sideport needle connected by a silicon tube to a bottle top dispenser (Sartorius, Germany) put on a 1 L glass bottle. The injection depth differed from the drilled depth to prevent overflow out of the drilled holes in the upper depth during the injections, as the tracer solutions infiltrated into the soil rather slowly. Each hole received 2 ml of the respective tracer solution, resulting in a total of 64 ml for the upper depth and 62 ml for the lower depth per subplot. A funnel was placed around the injection hole to prevent contamination of the vegetation with the tracer solution during tracer application.

Exactly 48 h after finishing the labeling of each subplot (20 to 21 April, 29 to 30 June and 29 to 30 September 2011), root crowns of three to five individual plants of each species present per plot were collected, cleaned and pooled by plant species and subplot. Three soil samples were taken at each subplot with a soil auger of 1 cm diameter (Eijkelkamp, The Netherlands) in nine soil depths (0–3, 3–6, 6–10, 10–15, 15–20, 20–23, 23–26, 26–30 and 30–40 cm). One soil replicate was taken very close to an injection point for the upper soil depth, one very close to an injection point for the lower depth, and one in between injection points. Soil samples in each depth were pooled, resulting in nine soil samples per subplot, covering the top 40 cm. All plant and soil samples were immediately placed into 12 ml glass vials (Labco Limited, UK), sealed with a cap and parafilm, kept cool in a cooling box and transported to a freezer within two hours. Samples were kept frozen until cryogenic water extraction. In total, 360 soil samples were taken and analyzed at each labeling campaign. In addition 197 plant samples were taken in April, 192 in June, and 193 in September. Due to the low water content of some plant samples, only 148, 136 and 145 samples were analyzed for each campaign, respectively.

### Laboratory analyses

Xylem water in root crowns and soil water were extracted for isotopic analyses using a cryogenic water extraction line [30] and measured with a TC/EA high-temperature conversion/elemental analyzer coupled with a DeltaplusXP isotope ratio mass spectrometer via a ConFlo III interface (Thermo-Finnigan, Bremen, Germany; see Werner et al. [36] for further information). Oxygen and hydrogen isotopic compositions of the water samples are given in δ notation measured as (R_Sample_/R_Standard_)—1, and expressed in ‰. R is the ratio of ^18^O to ^16^O or ^2^H to ^1^H of the sample or the standard. Our standard was a working control standard, regularly calibrated against international standards (V-SMOW, SLAP, GISP). The overall precision of the measurements was ± 0.09 ‰ for δ^18^O and ± 0.37 ‰ for δ^2^H.

### Data analyses

All statistical analyses and graphics were done with R 2.14.1 (R Development Core Team 2011). Mixed effects models were carried out by using the *lmer* function within the *lme4* package [37]. Prior to analyses, all data were log transformed to meet the assumptions for mixed effects models that require normally distributed within-group errors. The maximum likelihood method was used to estimate the variance components. Block, plot identity (nested within block) and species identity were treated as random factors. Analyses were started from a null model containing the random factors. Fixed factors and interactions between the fixed factors were entered stepwise. Likelihood ratio tests (*Χ* ^2^) were applied to compare models and to test for a significant improvement of the model after adding the fixed effects.

To compare whether the δ^18^O or δ^2^H values in the xylem water of the samples taken after the labeling differ from the background samples, mixed-effect models were carried out, including sample type (i.e., back ground sample or labeled sample) as fixed factor separately for each labeling campaign.

Enrichment of the xylem water was then identified by calculating the difference of δ^18^O or δ^2^H values of the samples taken after the labeling and the respective average value of the plant background samples for each labeling time. Finally, effects of species richness, number of functional groups and functional group identity (i.e., grasses, legumes, small herbs and tall herbs) on uptake of ^18^O- or ^2^H-enriched water were tested for each labeling campaign by adding the fixed factors in the following order: species richness (SR, log-linear), functional group richness (FR, linear), functional group identity (FG), and the interaction between SR and FG.

## Results

### δ^18^O and δ^2^H values of soil water

**Figure 1:**
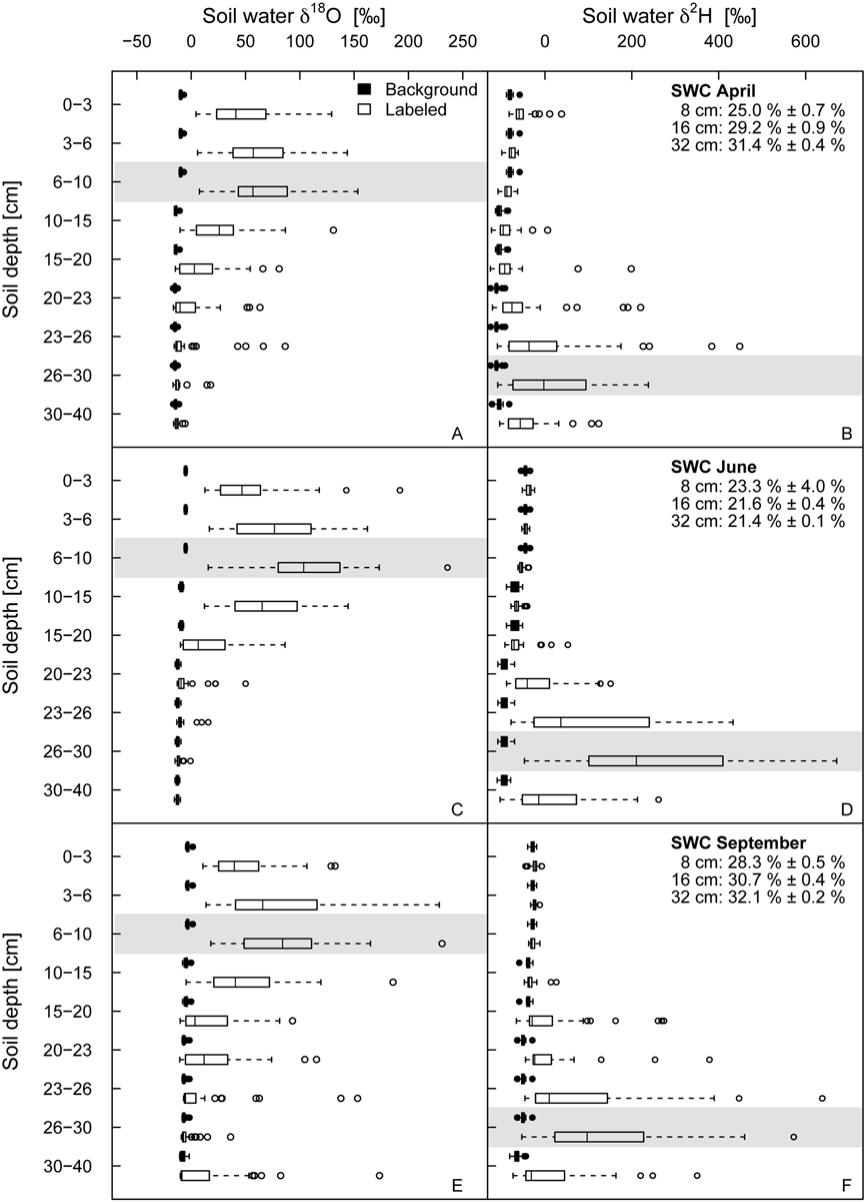
δ^18^O and δ^2^H values of soil water before and after the labeling. δ^18^O (A, C, E) and δ^2^H (B, D, F) values of soil water labeled with ^18^O-enriched water in upper soil depths and with ^2^H-enriched water in lower soil depths. Grey bands illustrate depths of tracer application. Data are given for the natural background soil as well as after the labeling at three different times in 2011 (April: A-B, June: C-D, September: E-F) in each case pooled for all species richness levels. Values of soil water content (SWC) in 8, 16 and 32 cm are given as mean ± 1 SD for the 4-day labeling and harvest campaigns.

Soil water in a depth of 6 to 10 cm, where the ^18^O-enriched water was injected, displayed average δ^18^O values across all species richness levels of 65.5 ‰ (SD ± 39.45 ‰) in April, 106.7 ‰ (SD ± 44.14 ‰) in June, and 85 ‰ (SD ± 45.64 ‰) in September (Fig. 1), highly enriched compared to the respective background values (δ^18^O_April_ = -9.67 ‰ (SD ± 1.15 ‰), δ^18^O_June_ = -5.08 ‰ (SD ± 0.83 ‰) and δ^18^O_September_ = -2.79 ‰ (SD ± 1.98 ‰). Similarly, soil water in a depth of 26 to 30 cm, where the ^2^H-enriched water was added, showed on average δ^2^H values of 16.9 ‰ (SD ± 99.32 ‰) in April, 262.6 ‰ (SD ± 206.21 ‰) in June, and 144 ‰ (SD ± 171.22 ‰) in September, highly above the background values (δ^2^H_April_ = -110.27 ‰ (SD ± 7.45 ‰), δ^2^H_June_ = -93.14 ‰ (SD ± 10.24 ‰) and δ^2^H_September_ = -50.12 ‰ (SD ± 7.61 ‰)). Soil layers above the target depths were enriched as well (Fig. 1), Mainly for δ^18^O, enrichment of soil water in layers below the target depth was also found at some plots. However, two distinct soil layers imitating two different water sources were achieved at all three campaigns.

During the course of the growing season, background δ^18^O and δ^2^H values increased by about 7 ‰ and 60 ‰ in the target depth (6–10 cm for ^18^O and 26–30 cm for ^2^H), driven by enhanced soil water evaporation at higher temperatures and changes in the isotopic composition of precipitation [38, 39].

### δ^18^O and δ^2^H values of xylem water

**Figure 2:**
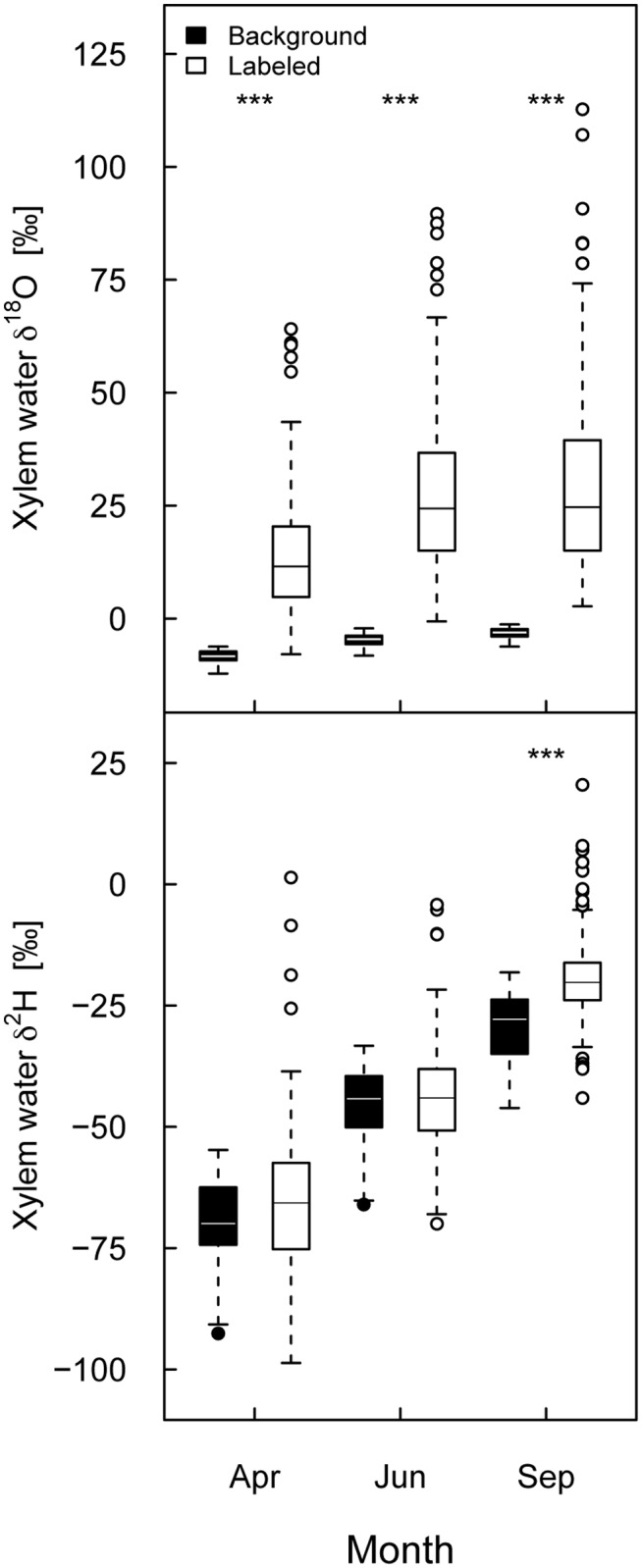
δ^18^O and δ^2^H values of xylem water before and after the labeling. δ^18^O (top) and δ^2^H (bottom) values are given for the background samples and the samples taken after the labeling at three different times (April, June, September 2011) in each case pooled for all species richness levels. Outliers (at δ^18^O = 141.7 ‰ and δ^2^H = 101.3 ‰ in June and at δ^18^O = 187.3 ‰ in September) were removed for reasons of clarity. Results of the corresponding mixed-effects models are given in the running text. Significant differences among the background samples and the samples taken after labeling are indicated with asterisks (with *** referring to *P* < 0.001).

Xylem water after the labeling and pooled over all species richness levels displayed average δ^18^O values of 14.24 ‰ (SD ± 14.21 ‰) in April, 28.88 ‰ (SD ± 21.01 ‰) in June, and 30.4 ‰ (SD ± 24.1 ‰) in September, well above the corresponding background values of -8.5 ‰ (SD ± 1.5 ‰) in April, -4.78 ‰ (SD ± 1.39 ‰) in June, and -3.35 ‰ (SD ± 1.27 ‰) in September. The δ^18^O values of the xylem water after the labeling were significantly higher than the δ^18^O values of the xylem water of the background samples at all three times (April: *Χ* ^2^ = 209.82, *P* < 0.001; June: *Χ* ^2^ = 220.37, *P* < 0.001; September: *Χ* ^2^ = 227.16, *P* < 0.001, Fig. 2). In contrast, δ^2^H values in the xylem water of the plants after the labeling did not differ significantly from background samples in April (*Χ* ^2^ = 0.87, *P* = 0.350) and June (*Χ* ^2^ = 1.19, *P* = 0.276), but in September (*Χ* ^2^ = 65.75, *P* < 0.001). While δ^2^H values of the xylem water of labeled plants were -65.37 ‰ (SD ± 14.75 ‰) in April, -43.13 ‰ (SD ± 16.78 ‰) in June and -19.16 ‰ (SD ± 9.02 ‰) in September, δ^2^H values of background plants were -69.81 ‰ (SD ± 9.71) in April, -45.6 ‰ (SD ± 8.05 ‰) in June, and -29.71 ‰ (SD ± 7.53 ‰) in September (Fig. 2).

The enrichment of xylem water in ^18^O, i.e., the difference between the average seasonal background δ^18^O value and the δ^18^O values of the samples taken after the labeling, ranged between 22.74 ‰ and 33.75 ‰ during the growing season, in comparison to the much larger enrichment in the soil water that ranged between 75.12 ‰ and 111.78 ‰ at 6 to 10 cm soil depth. However, the enrichment of xylem water in ^2^H ranged only between 2.47 ‰ and 10.55 ‰, despite a very large enrichment in the corresponding target depth of 26 to 30 cm soil depth (127.17 ‰ to 355.69 ‰), clearly indicating preferential water uptake in the upper soil depth.

**Figure 3:**
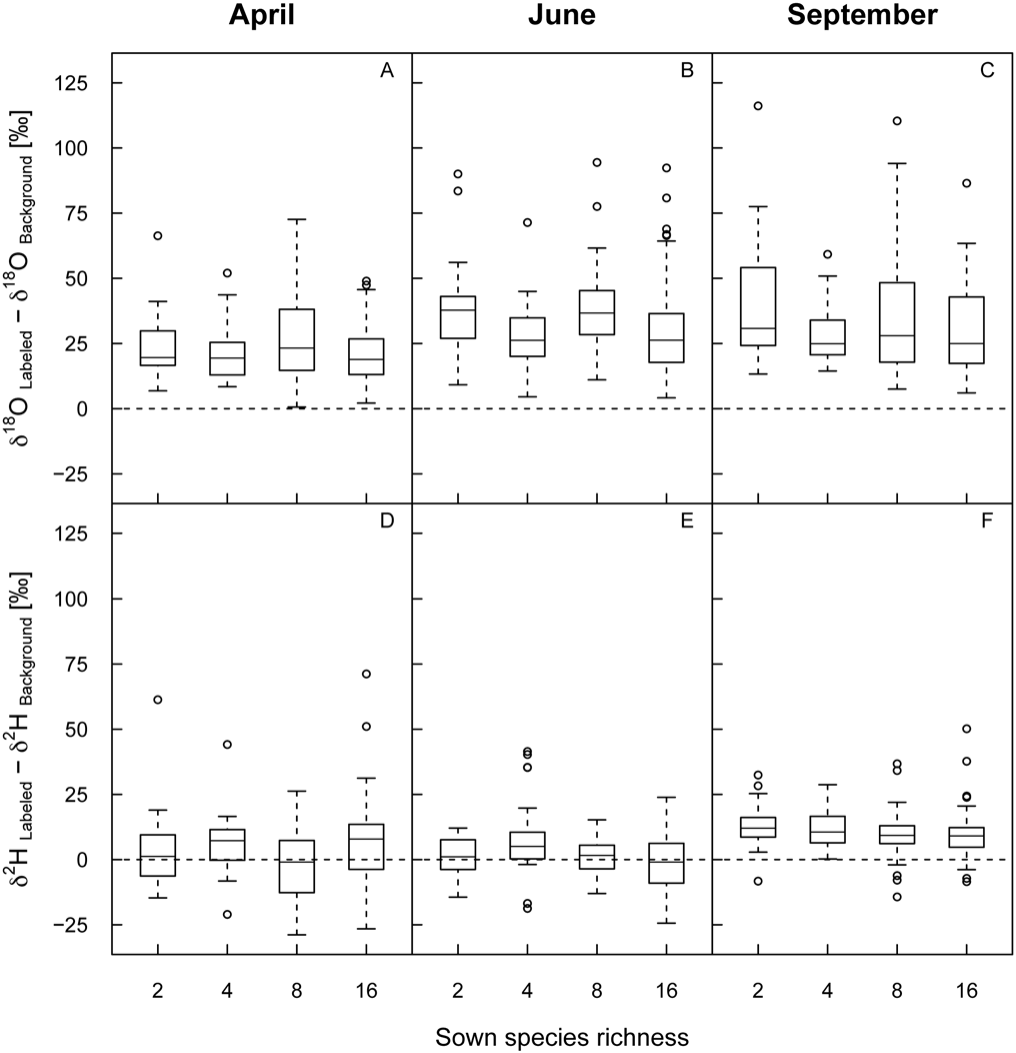
Enrichment of the xylem water in ^18^O or ^2^H along the sown species richness gradient. Enrichment was calculated as differences in δ^18^O (A-C) and δ^2^H (D-F) values in the xylem water after the labeling compared to the corresponding background at three different times (April, June, September 2011) separately for each species richness level. Outliers (at δ^18^O = 146.5 ‰ and δ^2^H = 146.9 ‰ in June and at δ^18^O = 190.6 ‰ in September) were removed for reasons of clarity. Results of the corresponding mixed-effects models are given in Table 1.

Enrichment of the xylem water in ^18^O or ^2^H was not affected by species richness or number of functional groups at any time (Table 1, Fig. 3). Functional groups only differed in their ^18^O enrichment in April, but not in June or September (*P_FG_* = 0.005, Table 1), with legumes displaying lower and small herbs slightly higher ^18^O enrichments compared to the other functional groups in April (legumes: δ^18^O = 11.61 ‰ (SD ± 8.14 ‰), small herbs: δ^18^O = 27.39 ‰ (SD ± 16.08 ‰), tall herbs: δ^18^O = 22.96 ‰ (SD ± 15.02 ‰), grasses: δ^18^O = 20.75 ‰ (SD ± 9.95 ‰). No difference among functional groups was found for ^2^H enrichment at any time.

**Table 1:** Summary of the mixed-effects model testing the effects of species richness, functional group number and functional group identity on xylem water enrichment in ^18^O and ^2^H (i.e., difference between samples taken after the labeling and the background samples).

	**April**				**June**				**September**			
	**δ^18^O**		**δ^2^H**		**δ^18^O**		**δ^2^H**		**δ^18^O**		**δ^2^H**	
	***χ^2^ ratio***	***P***	***χ^2^ ratio***	***P***	***χ^2^ ratio***	***P***	***χ^2^ ratio***	***P***	***χ^2^ ratio***	***P***	***χ^2^ ratio***	***P***
Species richness (SR, log-linear)	0.71	0.399	0.01	0.931	0.18	0.669	1.82	0.177	3.01	0.083	2.13	0.145
Functional group richness (FR, linear)	0.05	0.825	1.45	0.228	0.26	0.61	0.79	0.373	1.1	0.294	0.14	0.709
Functional group identity (FG)	12.94	**0.005**	3.79	0.285	6.11	0.106	2.13	0.546	2.62	0.453	5.82	0.121
SR x FG	0.19	0.801	1.6	0.66	2.37	0.499	1.73	0.63	0.85	0.837	2.58	0.461

Analyses were carried out for each campaign separately.

## Discussion

With the present study, we tested if plant communities of increased species or functional group richness exhibit increased spatial or temporal complementarity in water use compared to low diverse communities. Our results suggest that the main water uptake was from the top soil layers in all mixtures at all times, indicated by a considerable enrichment of xylem water in ^18^O (applied to the top soil layer) but not in ^2^H (applied to the deeper soil layer). We found no evidence for increased water exploitation from deeper soil layers with increasing species richness or functional group richness nor effects of functional group identity on spatial or temporal exploitation of soil water. Increased ^2^H enrichment of xylem water in September indicated uptake from the ^2^H enriched soil layer, but was not affected by increasing species or functional group richness. Thus, our results do not support the hypothesis of complementary water use as explanation for a positive biodiversity-ecosystem functioning relationship, neither spatially nor temporally.

The approach of our study was to create two distinct soil layers highly enriched in ^18^O (top soil layer) or ^2^H (deeper soil layer) to imitate two different water sources, which we generally achieved. However, in some plots, soil layers above (and below) the target depths were enriched as well (Fig. 1), most likely due to slow soil infiltration and percolation of the tracer solution when injected into the holes. A further reason might be hydraulic lift, although this has not been shown in grasslands yet [40]. The enrichment of some soil water samples below the target depth might have been caused by soil cracks or earthworm holes [41]. Furthermore, the enrichment of soil and xylem water in ^18^O and ^2^H was lower than intended. Although we aimed to achieve a homogenous distribution of the tracer solution by evenly distributing injection holes across the plots, horizontal infiltration and distribution of the tracer within the soil seemed to have been limited. Therefore, soil samples taken close to an injection point and in between injection points and then pooled by layer for each plot probably reflected a mix of enriched and unenriched soil water available for root uptake. However, since the experimental set-up was identical for all species richness levels, no bias was introduced, rendering the conclusion of preferential water uptake from the top soil layers as very robust.

Our experimental results, based on direct measurements of soil water use, contradict earlier studies that inferred water complementarity based on indirect approaches. For instance, Caldeira et al. [21] studied soil moisture patterns and plant δ^13^C in Mediterranean grasslands of varying species richness and interpreted lower foliar δ^13^C values of plants growing in mixtures than in monocultures as a result of more complete water use due to higher stomatal conductance rates. Verheyen et al. [20] considered complementary water use as the underlying mechanism for increased evapotranspiration with increasing species richness obtained from canopy surface temperature measurements. Van Peer et al. [42] reported increased water consumption with increasing species richness in heat stressed, container-grown artificial grasslands based on soil moisture measurements. However, lower δ^13^C values and thus higher stomatal conductance rates can also be the result of low light levels due to higher community biomass, which could in turn increase community evapotranspiration and lower canopy temperature. Thus, these indirect approaches cannot be used to unequivocally disentangle cause and effects.

On the other hand, studies using stable isotopes to directly test water uptake among coexisting species found strong evidence for water partitioning, but typically in semi-arid ecosystems, where water availability is naturally limited [12, 22, 26, 32, 43–45]. However, none of these studies tested different species richness levels. Thus, spatial niche differentiation seems more likely to allow for coexistence when water in upper soil layers is scarce than under conditions when water is not a limiting resource (see soil water content given in Fig. 1). Our results are also in line with the stress-gradient hypothesis, assuming low levels of competitive interactions in plant communities under low levels of stress [46]. Under such conditions, water availability is closely linked to nutrient availability, which is higher in upper than in deeper soil layers of the Jena Experiment (personal communication Markus Lange, see also [47–49]), thus favoring the development of a shallow rooting system [50], even along a diversity gradient.

Furthermore, complementarity in belowground resources use (water, nutrients) is thought to result from an increased dissimilarity of rooting depths among species with increasing species richness. Hence, vertical root biomass distribution is expected to change in favor of increasing root biomass also in deeper soil layers with increasing species richness. However, Ravenek et al. [51] did not find any shifts in relative root distribution along the vertical soil profile with increasing species richness or in plots with different functional group composition. The ratio of standing root biomass among different soil layers remained quite similar, despite a significant increase in total standing root biomass at higher species richness levels. These results give further support for a lack of vertical niche differentiation with increasing species richness, but rather show preferential resource uptake from the upper soil layers independent of species or functional group richness.

Clear experimental evidence for complementarity is also scarce for other soil resources, e.g., nitrogen. In two grassland studies, both conducting ^15^N labeling experiments, neither spatial nor temporal complementarity of nitrogen uptake was found in more diverse grasslands compared to low diverse grasslands [52, 53]. In both studies, the main nitrogen uptake was from the top soil layer (upper 3 cm).

Ecosystem processes have been found to be highly influenced by the functional group composition rather than by species richness alone [54, 55]. Kahmen et al. [52] observed significant differences in nitrogen uptake among different functional groups (legumes, tall herbs, legumes, small herbs), irrespective of species richness level. In our study, differences in water uptake among functional groups were not significant except for April 2011, the very start of the growing season when growth commences. Based on information derived from the literature, small herbs are assumed to have shallower roots than tall herbs, grasses and legumes in the Jena Experiment [56, 57], but roots of most species cover the depths studied with our labeling approach and root characteristics vary greatly among species within functional groups. This variation may explain the lack of a consistent functional group effect on water uptake patterns in our experiment.

In conclusion, our results suggest no increased complementarity in water use with increasing species richness. The main water uptake from the top soil layer is consistent with observed rooting patterns as well as with results on nitrogen uptake found in other temperate grasslands. If complementarity in water use differs between systems adapted to low vs. high water availability remains to be seen. Furthermore, since plant species are often limited by multiple resources and differ in their resource requirements [10, 58], complementarity not only for a single resource, but for multiple resources might be the mechanism to explain the positive effects of high plant species richness on ecosystem processes.

## Supporting Information

S1 Fig Application scheme of the tracer solution. (DOCX)

S1 Table List of mixtures used for the tracer experiment. (XLSX)
