# Supplementary material for: No Evidence of Complementary Water Use along a Plant Species Richness Gradient in Temperate Experimental Grasslands
Source: PLoS One. 2015 Jan 14;10(1):e0116367. doi: 10.1371/journal.pone.0116367 (PMC4294640; doi:10.1371/journal.pone.0116367)
Supplement: S1 Fig — (DOCX) [file pone.0116367.s001.docx]

**Figure S1. Application scheme of the tracer solution.**
